# Statistical implication analysis: a novel approach to understand the reciprocal relationships between outcomes in early psychosis

**DOI:** 10.1017/S0033291724001430

**Published:** 2024-09

**Authors:** Philippe Golay, Lilith Abrahamyan Empson, Nadir Mebdouhi, Caroline Conchon, Vincent Bonnarel, Philippe Conus, Luis Alameda

**Affiliations:** 1 General Psychiatry Service, Treatment and Early Intervention in Psychosis Program (TIPP–Lausanne), Lausanne University Hospital and University of Lausanne, Lausanne, Switzerland; 2 Department of Psychiatry, Community Psychiatry Service, Lausanne University Hospital and University of Lausanne, Lausanne, Switzerland; 3 Faculty of Social and Political Science, Institute of Psychology, University of Lausanne, Switzerland; 4 Department of Psychosis Studies, Institute of Psychiatry, Psychology and Neuroscience. King's College of London, London, UK; 5 Departamento de Psiquiatria, Centro Investigacion Biomedica en Red de Salud Mental (CIBERSAM); Instituto de Biomedicina de Sevilla (IBIS), Hospital Universitario Virgen del Rocio, Universidad de Sevilla, Sevilla, Spain

**Keywords:** early psychosis, outcome, schizophrenia, statistical implication

## Abstract

**Background:**

Patients can respond differently to intervention in the early phase of psychosis. Diverse symptomatic and functional outcomes can be distinguished and achieving one outcome may mean achieving another, but not necessarily the other way round, which is difficult to disentangle with cross-sectional data. The present study's goal was to evaluate implicative relationships between diverse functional outcomes to better understand their reciprocal dependencies in a cross-sectional design, by using statistical implication analysis (SIA).

**Methods:**

Early psychosis patients of an early intervention program were evaluated for different outcomes (symptomatic response, functional recovery, and working/living independently) after 36 months of treatment. To determine which positive outcomes implied other positive outcomes, SIA was conducted by using the Iota statistical implication index, a newly developed approach allowing to measure asymmetrical bidirectional relationships between outcomes.

**Results:**

Two hundred and nineteen recent onset patients with early psychosis were assessed. Results at the end of the three-years in TIPP showed that working independently statistically implied achieving all other outcomes. Symptomatic and functional recovery reciprocally implied one another. Living independently weakly implied symptomatic and functional recovery and did not imply independent working.

**Conclusions:**

The concept of implication is an interesting way of evaluating dependencies between outcomes as it allows us to overcome the tendency to presume symmetrical relationships between them. We argue that a better understanding of reciprocal dependencies within psychopathology can provide an impetus to tailormade treatments and SIA is a useful tool to address this issue in cross-sectional designs.

## Introduction

Early intervention is now widely considered as the standard approach to treating psychotic disorders. One of the major challenges of early intervention is to reduce the symptom disability gap in the early course of the disease, and to reduce the long-term disability (Birchwood, Todd, & Jackson, 1998). Despite the recognized effectiveness of early intervention programs, not all patients reach favorable symptomatic or functional outcomes at the end of their program (Edwards et al., 2002; Golay et al., 2020; 2021; Robinson, Woerner, McMeniman, Mendelowitz, & Bilder, 2004). Indeed, in a systematic review and meta-analysis of long-term remission and recovery from first-episode psychosis, about 58% of patients achieved remission and 38% recovery (Lally et al., 2017). While to some extent larger than previously thought, those estimates highlight that there remains a significant proportion of patients who have persistent psychotic symptoms and experience disability, suffering, and family burden. Recent large representative studies of early psychosis (EP) patients showed that functional impairment was highly prevalent in patients with EP, with rates of functional impairment ranging between one-third and half of EP (Chang et al., 2018; Golay et al., 2021; Hall, Holton, Öngür, Montrose, & Keshavan, 2019; Hodgekins et al., 2015). These studies also revealed the heterogeneous courses of socio-occupational functioning during EP, with distinct functional trajectories. In particular, Chang et al. (2018) reported that close to half of the patients displayed a persistently poor trajectory over three years, indicating that socio-occupational impairment had been an unmet therapeutic need.

In a recent study, we showed that symptomatology had a high degree of specificity and that specific symptoms accounted for more than 70% of the variance of psychiatric symptomatology assessments (Golay et al., 2022). We also argued for a staging approach that should evolve to fully embrace this multidimensional organization of psychopathology and its outcomes. In this line, a distinction between ‘disease progression’ (worsening of the syndrome itself) and ‘disease extension’ (spreading of the syndrome to have wider reaching effects on multiple outcomes) was suggested (Carpenter et al., 2019). Because patients can respond differently to early interventions for their psychosis, diverse symptomatic and functional outcomes can be distinguished and may be reached at different times.

### Standard approaches and the need for new alternatives

In the study of developmental processes, success on one task may imply success on another, but not necessarily vice versa. One skill may precede the development of another over time. Observation of the latter skill will then almost always be accompanied by observation of the former. This is what we can label as ‘implication’. Achieving outcomes could also be considered from the point of view of implication: achieving one positive outcome may mean achieving another, but not necessarily the other way round. Unfortunately, in the ubiquitous associative framework, the order of variables is irrelevant. Most measures of associations between variables, such as the correlation coefficient or the Chi-square statistic are symmetrical: these coefficients give identical results if the order of the variables is reversed. The same holds true for most psychometric networks analysis models: Unless they are estimated on longitudinal data, they estimate the strength of undirected relationships between variables. They are therefore unable to give a satisfactory account of many developmental phenomena. At best, they could reveal a positive manifold of correlations between variables, suggesting patients that achieved some positive outcomes were more likely to achieve other outcomes. Such measures of association will however fail to satisfactorily account for complex symptoms and outcomes reciprocal interactions (Golay et al., 2022). In this situation, there is a need to rely on novel methods that go beyond these major limitations. Statistical implication analysis (SIA) was coined by Gras, Suzuki, Guillet, and Spagnolo (2008). Further work by Noël (2017, 2021) provide some answer to this with the computation of full-information asymmetric indices. The implication from A to B can be described as a probabilistic relationship where B is very often observed in the presence of A and where counterexamples are rare. This implicative framework could be considered as another manner to study relationsips between variables in a more complex yet subtle asymmetrical way. The present study's goal was to, for the first time, apply a SIA framework to a psychiatry setting aiming at highlighting reciprocal relationships between variables; and to by doing so, better understanding the mutual relationships of diverse functional outcomes in early psychosis.

## Material and methods

### Participants

The Treatment and Early Intervention in Psychosis Program (TIPP) is a specialized EP program run by Lausanne University Hospital's Department of Psychiatry, in Switzerland (Baumann et al., 2013). Participants' inclusion criteria are: being aged from 18 to 35, living in the hospital's catchment area (population about 350 000), and meeting the criteria for psychosis as defined by the ‘psychosis threshold’ subscale in the Comprehensive Assessment of At-Risk Mental States (CAARMS) instrument (Yung et al., 2005). Here psychotic disorder threshold is defined as having frank psychotic symptoms such as delusions, hallucinations, and thought disorder persisting for longer than one week, with a frequency of at least 3–6 times a week for longer than 1 h each time or daily for less than 1 h each time. This is a standard and widely used criterion for first episode psychosis threshold (Nelson, Yung, Markulev, & Nicoli, 2014).

Patients with drug-induced brief psychotic states, organic brain disease, an IQ < 70, or those on antipsychotic medication for more than six months are referred to another program. The TIPP paradigm of care is based on the principles of both case management interventions and assertive community treatment. Over a three-year period, case managers are available to each patient up to twice a week. Patients are seen at least 100 times over the three-year program, primarily by their case manager but also by a resident physician or an intern in psychiatry. A consultant psychiatrist supervises each case.

All patients treated within the TIPP are assessed at baseline. A specially designed questionnaire (the TIPP Initial Assessment Tool: TIAT; available online (Service of General Psychiatry DoP, 2021)) is completed for all patients enrolled in the program by case managers. It allows assessment of demographic characteristics and past medical history. Follow-up assessments exploring various aspects of treatment and co-morbidities as well as evolution of psychopathology and functional level are conducted by a psychologist and by case managers during the 36 months of treatment.

The authors assert that all procedures contributing to this work comply with the ethical standards of the relevant national and institutional committees on human experimentation and with the Helsinki Declaration of 1975, as revised in 2008. This study was approved by the Human Research Ethics Committee of the Canton of Vaud (CER-VD; protocol #2020-00272). The data generated by the follow-up of all patients were used in the study if the latter did not explicitly object to the use of their data for research purposes. Only four patients refused the use of their clinical data for research. Patients were included in the present analysis only if data on every outcome was available.

### Clinical assessments

Detailed evaluation of past medical history, demographic characteristics, as well as symptoms and functioning were performed by case managers (CM) and a psychologist, through semi-structured interviews and a questionnaire. Case managers and an experienced psychologist performed detailed evaluations of patients’ using interviews and the TIAT questionnaire. The DUP was defined as the time between the onset of the psychotic symptoms defined by the CAARMS and admission to the TIPP. Patients’ socioeconomic statuses were subdivided into low, intermediate, and high (Chandola & Jenkinson, 2000). Diagnosis is the result of an expert consensus and is based on the following elements: (1) Diagnosis based on DSM-IV criteria reported by a treating psychiatrist in all medical documents and at the end of any hospitalization; (2) longitudinal assessment by psychiatrist, psychologist and clinical case manager over the 3 years of treatment. The consensus diagnosis procedure is realized by a senior psychiatrist and the senior psychologist in charge of scale-based assessment over the treatment period. They both review the entire file once after 18 months and again after 36 months (or at the end of treatment) and conduct a diagnostic process discussing any unclear issue with the case manager. In this study, we considered the latest diagnostic consensus available.

### Outcomes

Functional recovery after three years was defined as a Global Assessment of Functioning (GAF) score >60. Symptomatic remission at the end of the program was defined by the last Positive And Negative Syndrome Scale assessment score in the last year of the program, following Andreasen's Criteria (mild or lower (⩽3) score on the following items: delusion, unusual thought content, hallucinatory behavior, conceptual disorganization, mannerisms, blunted affect, social withdrawal, and lack of spontaneity; Andreasen et al., 2005). Functional characteristics at the end of the program were assessed using the Modified Vocational Status Index and the Modified Location Code Index (MVSI & MLCI; Tohen et al., 2000a). Patients were considered as *living independently* at the end of the program based on their MLCI score (head of household or living alone, living with a partner or peers, or living with their family with minimal supervision). Patients were considered as *working* at the end of the program based on the MVSI (in paid or unpaid, full- or part-time employment, being an active student in school or university, head of household with an employed partner (homemaker), or a full or part-time volunteer).

### Statistical analysis: statistical implication analysis (SIA)

We estimated whether achieving some outcomes implied achieving other outcomes. Oriented dependencies of the different outcomes were estimated using the Iota statistical implication index for dichotomous variables (Noël, 2021). Unlike symmetrical indices like correlation coefficients, where the order of variables does not play any role, asymmetrical bidirectional relationships can also be distinguished. An example of an asymmetrical relationship might be a child's ability to walk in relation to tying his shoes. Knowing how to tie one's shoes will almost always imply knowing how to walk, whereas knowing how to walk will not systematically imply knowing how to tie one's shoes. *ι*_A⇒B_ allows to estimate whether the presence of A implies that B will also be present while *ι*_B⇒A_ allows to estimate whether the presence of B implies that A will also be present. Naturally, both relationships can take different values. *ι*_A⇒B_ varies from −∞ to +∞ with positive values showing evidence in favor of the implication from A to B, 0 representing uncertainty, and negative values showing evidence against implication (Noël, 2021). As this approach is new, there is not yet a clearly defined and consensual coefficient value that may indicate whether the implication could be considered as small, medium, or large for example. Nevertheless, the coefficients can be tested for statistical significance and can be compared, a higher coefficient indicating a stronger implication. The Iota implication index was chosen because it is superior to other coefficients that estimate the implication strength based on the rarity of counterexamples only. In contrast, the Iota coefficient is also able to exploit counter positive information (for instance when ‘A implies B’, the counter positive is ‘not B implies not A’) (Noël, 2021). Statistical significance of the Iota coefficient was fixed at the 0.05 level and was determined using bootstrap. The 95% confidence interval on the index was computed and checked whether it contained zero. All statistical analyses were performed using IBM SPSS 27 and the ‘boot’ package for R (Canty & Ripley, 2016).

## Results

The final sample (Table 1) consisted of 219 EP patients (Mean age = 24.92; s.d. = 4.74) that were consecutively included if they had complete 36-months outcome data. It included a majority of male (66.7%) and among these patients, 56.6% met diagnostic criteria for schizophrenia, 12.3% for schizoaffective disorder, 11.0% for schizophreniform or brief psychotic disorder, 6.8% for bipolar disorder with psychotic features, 3.2% for depression with psychotic features, and 10.0% for other psychotic disorders.

**Table 1.** Demographic patients’ characteristics and outcomes

	*N* = 219
Age in y, M (s.d.)	24.92 (4.74)
Sex, male, % (*N*)	66.7 (146)
SES, % (*N*)	
Low	18.7 (41)
Intermediate	44.3 (97)
High	37.0 (81)
DUP in days, median (IQR)	84.00 (464.00)
Age of onset in y, M (s.d.)	23.47 (5.18)
Diagnosis, % (*N*)	
Schizophrenia	56.6 (124)
Schizophreniform/BPE.	11.0 (24)
Schizo affect if disorder	12.3 (27)
Major depression^ª^	3.2 (7)
Biopolar disorder^ª^	6.8 (15)
Others	10.0 (22)
Symptomatic response (PANSS), % (*N*)	53.4 (117)
Working at baseline (MVCI), % (*N*)	29.2 (64)
Functional recovery (GAF), % (*N*)	54.3 (119)
Independent living at the end of the program (MLCI), % (*N*)	66.2 (145)
Working at the end of the program (MVCI), % (*N*)	33.3 (73)

*Note*. IQR, interquartile range; BPE, brief psychotic episode.

^a^With psychotic features.

MLCI, Modified Location Code Index; MVCI, Modified Vocational Status Index.

**Figure 1.**
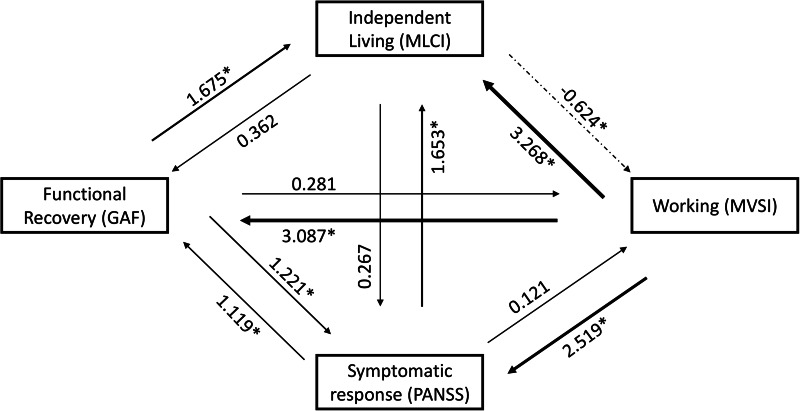
Statistical implication analysis of the different outcomes at the end of the program with the Iota index for dichotomous variables (Noël, 2021).

Results of the SIA revealed that working independently at the end of the program statistically implied achieving all other positive outcomes (Fig. 1). Symptomatic and functional recovery reciprocally implied one another. Living independently did not significantly imply symptomatic and functional recovery and did not imply working. In other words, when achieved, living independently was not as regularly observed in conjunction with having a professional activity.

Because working independently at the end of the program statistically implied achieving all other positive outcomes, we conducted a post-hoc analysis to verify whether patients who worked independently or not at the beginning of the program had similar results (Table 2). Working did not significantly imply functioning recovery with patients that were already working at baseline although working still implied independent living and symptomatic response for these patients.

**Table 2.** Post-hoc analysis focused on independent work at the end of the program in function of independent work at baseline

Subgroup	Variable A	Variable B	Iota statistical implication index (Noël, 2021)	
			*ι*_A⇒B_	*ι*_B⇒A_
*Not working at baseline*	Working	Independent living	4.259*	−0.777
*Working at baseline*	Working	Independent living	2.015*	−0.491
*Not working at baseline*	Working	Symptomatic response	4.118*	0.001
*Working at baseline*	Working	Symptomatic response	1.674*	0.939
*Not working at baseline*	Working	Functional recovery	3.269*	0.141
*Working at baseline*	Working	Functional recovery	1.015	−0.268

Note. *Significant at 0.05 level. 51.6% of patients working at baseline are working at the end of the program. 25.5% of patients not working at baseline are working at the end of the program.

## Discussion

We used the SIA method to better understand reciprocal dependencies between various outcome dimensions in early psychosis. To the best of our knowledge, this is the first study applying this novel approach to the understanding of psychiatric outcomes. Unlike classical symmetrical associative measures, our analysis revealed a more complex picture of association between different symptomatic and functional outcomes. First, there is a strong statistical implication between working independently at the end of the program and other outcome domains. Second, symptomatic and functional recovery reciprocally strongly implied one another. Finally, independent living at the end of the program only weakly implied functional and symptomatic recovery and did not imply working independently.

Return to independent activity seems to be in connexion with all other facets of outcome. Indeed, patients who hold an independent activity at the end of treatment strongly tend to have recovered in all other dimensions although working did not significantly imply functioning recovery with patients that were already working at baseline. Considering that the type of analysis we applied does not allow to infer a strict causality between the various dimensions that are interconnected, two interpretations of the results can be proposed. First, it is likely that the condition for a return to independent activity is to have recovered in all other dimensions. However, a second interpretation would rather suggest that return to work contributes to the recovery in all other dimensions. This is to some degree what underlies strategies such as supported employment: returning to work may have a normalizing effect, an impact on self-esteem and on the motivation for treatment and could therefore lead to recovery in other dimensions. This finding cannot be solely accounted for by the fact that patients who worked independently at the end of the program were already working at the beginning of the program. Indeed, the subgroup analysis revealed that this pattern held true for all patients regardless of their work status at the beginning of the program.

Many papers have shown, especially in affective psychosis patients (Conus et al., 2006; Conus, Macneil, & McGorry, 2014) that functional and symptomatic recovery seem to evolve distinctly, many first-episode mania displaying a swift reduction of manic and psychotic symptoms but maintaining poor functional recovery; the same seem to hold true in non-affective psychoses (Tohen et al., 2000b). Our data suggest however that these two dimensions are tightly and reciprocally linked to one another. This is in phase with the frequent observation that, although aiming at measuring conceptually distinct yet related dimensions, scores on global assessment of functioning, including both symptoms and function (GAF) and Social and Occupational Functioning Assessment Scale (SOFAS), assessing functional level only) are empirically very highly correlated when measured with the same patients (Alameda et al., 2017). A simple explanation for this tight relationship could be that symptomatic recovery is most of the time a condition for achieving return to functional recovery and vice-versa.

Independent living only weakly implied symptomatic and functional recovery and did not imply working at all. In other words, living independently seems possible even if patients still have symptoms and do not have an independent activity. This may be linked to the fact that approach such as Housing First (Macnaughton et al., 2015) have been implemented in our region, which favor independent living even when patients are still quite ill, in order to avoid precarity and contribute to a better engagement with patients (Garcia Gonzalez De Ara et al., 2017). The support of intensive home support via mobile teams certainly plays a role in this observation as well (Bonsack, Adam, Haefliger, Besson, & Conus, 2005). This asymmetrical relationship between living independently and working is also to be put in perspective with previous studies showing that both domains of functioning are relatively independent and distinct constructs, the former being more affected by social cognition and trauma-related domains (Christy et al., 2023; Fares-Otero et al., 2023), the latter more dependent of neurocognitive abilities (Alameda et al., 2015).

From a clinical standpoint, our results encourage us to think more about the possible interactions and reciprocal relationships between outcomes rather than a list of diverse desirable outcomes to achieve. They also confirm the important role working independently may play in recovery. This is especially worth of attention because the return to independent activity may frequently prerequisite that patients have already recovered in other dimensions.

Regarding potential limitations, implication was defined as ‘statistical implication’ in the sense that counter examples were rare. This is distinct from perfect implication where counterexamples do not exist. Additionally, statistical implication is to be distinguished from causality because implication may be embedded in a complex network of dependencies (Noël, 2021). Second, this approach being novel, there is not yet a consensus established over the long term and based on various studies to accurately quantify the magnitudes of the implication estimates. Third, because of the naturalistic nature of the cohort, some patients of our representative clinical sample of patients could not be included because data on one or several outcomes was not available. Fourth, similar analysis using other measures of outcome should be conducted to validate them on a larger and different sample. Fifth, indicators may partially overlap (for instance GAF and occupational outcomes). With a classical approach, this may inflate symmetrical indices like correlations. Given our approach considers the asymmetrical orientation of the dependencies, we believe that the impact of this overlap is limited. Sixth, some outcomes were dichotomized as the statistical procedure requires variables to be binary. Seventh, the approach used is transversal which is easily applicable in a variety of scenarios but there may still be some research questions where a longitudinal design would be better suited.

## Conclusion

SIA and the concept of oriented dependencies is an interesting way of thinking because it allows us to overcome the tendency to presume relationships between variables in a symmetrical way. This is especially useful when we are using cross-sectional data. This paper illustrates that the SIA framework can be a highly valuable tool to provide some insights into the issue of reciprocal causation between outcomes in psychiatric settings, when prospective data is sometime very difficult to collect. Understanding mutual dependencies between outcomes could help clinicians to improve treatments. From a clinical standpoint, our findings suggest that clinicians should be aware that reciprocal interactions between symptoms and outcomes may contribute to transform very narrow and specific disorders into ultimately more general and broad clinical concepts. We argue that a better understanding of oriented dependencies within psychopathology can be seen as an opportunity to more tailormade treatments. In addition, while our study focused on implication regarding functional and symptomatic outcomes in recovery, SIA can be a very useful approach to grasp intricate implications in other complex contexts, where symptomatic, environmental, and sociocultural aspects are at stake (e.g. urban living and mental health or more broadly environmental psychiatry) thus contributing to a more situated understanding of phenomena and tailoring therapeutic interventions.
